# Enthalpy Relaxation of Polyamide 11 of Different Morphology Far Below the Glass Transition Temperature

**DOI:** 10.3390/e21100984

**Published:** 2019-10-10

**Authors:** René Androsch, Katalee Jariyavidyanont, Christoph Schick

**Affiliations:** 1 Interdisciplinary Center for Transfer-oriented Research in Natural Sciences, Martin Luther University Halle-Wittenberg, 06099 Halle/Saale, Germany; katalee.jariyavidyanont@iw.uni-halle.de; 2 Institute of Physics and Competence Center CALOR, University of Rostock, Albert-Einstein-Str. 23–24, 18059 Rostock, Germany; christoph.schick@uni-rostock.de; 3 Butlerov Institute of Chemistry, Kazan Federal University, 18 Kremlyovskaya Street, Kazan 420008, Russia

**Keywords:** polyamide 11, enthalpy relaxation, crystallinity, fast scanning chip calorimetry

## Abstract

Polyamide 11 (PA 11) samples of different supermolecular structure, including the crystal-free glass and semi-crystalline PA 11 of largely different semi-crystalline morphology, were prepared by fast scanning chip calorimetry (FSC). These samples were then annealed at different temperatures well below the glass transition temperature *T*_g_. The main purpose of the low-temperature annealing experiments was the calorimetric detection of mobility of chain segments at temperatures as low as −40 °C (≈*T*_g_ − 80 K) where still excellent impact resistance is predicted. It was found that annealing PA 11 at such low temperature, regardless the thermal history and supermolecular structure including crystallinity as well as crystal shape and size, permits distinct enthalpy relaxation at rather short time scale with the structural changes reverting on subsequent heating as detected with pronounced sub-*T*_g_-enthalpy-recovery peaks. The main glass transition, associated to large-amplitude segmental mobility, as well as relaxations at temperatures only slightly below *T*_g_ are even more distinctly sensitive to the crystal morphology. In contrast to spherulitically grown lamellar crystals, presence of high-specific-surface area nanometer-sized ordered domains causes a shift of the glass transition temperature of the amorphous phase to higher temperature, proving stronger coupling of ordered and amorphous phases than in case of lamellae. In addition, the increased coupling of the crystalline and amorphous phases slows down the cooperative rearrangements on annealing the glass slightly below *T*_g_. The performed study contributes to further understanding of the spectrum of structural relaxations in PA 11 including the effect of presence of crystals. Enthalpy relaxation and consequently the reduction of entropy at temperatures slightly below *T*_g_ strongly depends on the semi-crystalline morphology, while an only minor effect is seen on low-temperature annealing at *T*_g_ − 80 K, possibly indicating different molecular mechanisms for the processes occurring in both temperature ranges. The low-temperature process even seems proceeding in the crystalline fraction of the material.

## 1. Introduction

Polyamide 11 (PA 11) is an important thermoplastic material produced from short-term renewable castor oil, gaining increasing attention since it does not harm the environment like consumption of non-renewable crude oil. Due to its balanced property profile such as good chemical resistance, low oxygen- and hydrocarbon permeability, excellent low-temperature impact strength, or high thermal stability, it has found many industrial applications. These include off- and onshore oil and gas pipes, hydraulic and pneumatic hoses, electrical cable sheathing, sporting goods, or, related to its piezoelectricity, electronic-device applications [1,2,3,4]. PA 11 products are typically semi-crystalline, containing up to about 30% crystals, owing to its rather high rate of melt-crystallization. The critical cooling rate to suppress melt-crystallization and fully vitrify the melt at its glass transition temperature (*T*_g_) of around 40 °C is between 500 and 1000 K/s. Slower cooling allows crystallization, however, with the final semi-crystalline morphology strongly depending on the exact crystallization conditions. It was found that crystallization at low supercooling of the melt proceeds via heterogeneous crystal nucleation, leading to formation of lamellar α-crystals and spherulites while crystallization at high supercooling of the melt, at temperatures below about 110 °C, proceeds via homogenous nucleation and non-spherulitic growth of a nodular mesophase [4,5,6,7]. Such temperature-controlled change of the pre-dominant nucleation mechanism is observed for many polymers, [8,9,10] including further representatives of the polyamide family such as PA 6 [11,12], PA 66 [13], or PA 12 [14].

PA 11 typically is melt-processed by extrusion, blow-molding, injection-molding, rotomolding, but also 3D printing, and laser sintering, involving rather fast solidification during cooling and the generation of a large variety of unstable or metastable non-equilibrium structures [15,16,17,18]. Structural changes towards equilibrium may involve both the crystalline and amorphous phases, and often lead to a change of properties, requiring research for its quantification and understanding. Such irreversible changes of structure include enthalpy relaxation of the amorphous phase, crystallization of the amorphous phase, and reorganization of crystals, with these processes briefly described below.

A thermodynamically non-equilibrium amorphous structure is obtained on cooling the equilibrium liquid phase to below the equilibrium melting temperature *T*_m, 0_ of the inherently crystallizable system, being in case of PA 11 203 °C [19] or 220 °C [20]. However, the structure of the supercooled non-equilibrium liquid below *T*_m, 0_ apparently adjusts instantaneously on variation the temperature due to the short relaxation time of the order of magnitude of picoseconds [21]. As such, supercooled liquids are considered metastable, that is time-independent, unless crystal nucleation and growth occurs. Metastability, at least within a certain timeframe defined by the relaxation kinetics, is lost on vitrification of the supercooled liquid phase on further cooling the system to below *T_g_*, leading to the formation of an initially thermodynamically unstable glass [22,23]. Due to constraints imposed by the reduced free volume between molecular segments, structural relaxation of the system by changes of conformations of covalent bonds distinctly slows down, allowing its recognition at experimentally assessable time scales well above milliseconds, even millions of years [23,24,25,26]. Relaxation processes in unstable polymer glasses, as well as glasses of other classes of materials, include its densification towards a final state defined by the density/free volume and enthalpy of the corresponding liquid at identical temperature. Such relaxation occurs by both cooperative rearrangement of molecular segments at the nanometer-length scale but also non-cooperative changes of local chain conformations at the sub-nanometer scale, e.g., depending on temperature [27,28]. Importantly, though connected with decreases of the enthalpy and entropy of the system, these relaxation processes do not involve the formation of a new phase, as would be the case upon crystallization. The decrease of the free volume during glass relaxation has enormous impact on properties of polymeric materials as it may cause detrimental changes of, e.g., mechanical or transport properties, often denoted as physical aging [29,30,31,32,33].

Further processes occurring in non-equilibrium amorphous phases, in both the supercooled liquid and the glass, leading to a decrease of Gibb’s enthalpy towards equilibrium, are crystal nucleation and growth. Focusing on the glassy state, being in foreground in this manuscript, quantitative analysis of the kinetics of glass-crystallization in polymers recently became possible with the opportunity to prepare glasses of well-defined cooling history and to analyze efficiently the progress of structural changes on annealing the glass using fast scanning chip calorimetry [34]. A main conclusion derived from recent, tailored glass-relaxation- and -crystallization-experiments in polymers is the rather strict sequence of enthalpy relaxation, homogeneous crystal nucleation, and crystal growth [35,36,37,38,39,40]. It is explained such that the cooperative rearrangements of highly mobile short molecule segments at the length scale of few nanometers during enthalpy relaxation suppress growth of stochastically appearing nuclei to supercritical size, rather than lead to their disappearance. The interplay between enthalpy relaxation and crystal nucleation and growth in glasses has been confirmed for several polymers [35,36,37,38,39,40] but also for small inorganic molecules [41]. Worth noting, analysis of the temperature dependence of the kinetics of homogeneous crystal nucleation revealed that nucleation is fastest slightly above *T*_g_ and that nucleation is not affected by the main glass transition, that is, at temperatures around *T*_g_ nuclei formation requires segment mobility at a length scale shorter than freezing at the glass transition [8,9,37,42,43].

Besides enthalpy relaxation of the amorphous glass, crystallization of the supercooled liquid or glass in absence or presence of already existing crystals, further structural changes driving a decrease of Gibbs enthalpy of the system involve an increase of the stability of crystals, commonly described as crystal reorganization. Crystal reorganization typically occurs at temperatures close to their stability limit, that is, their melting point, and includes processes like lamellar thickening to decrease the surface-to-volume ratio, or healing of lattice defects. In this work, crystal reorganization is out of the scope, with further information available in the literature [44,45,46,47,48,49,50].

The present study focusses on changes of structure of PA 11 at temperatures far below *T*_g_, being also important for practical reasons. The temperature range of application of this particular material includes ambient and sub-ambient temperatures, with superior low-temperature impact resistance reported being evident down to −40 °C [51], which is around 80 K below the glass transition temperature. Impact strength, toughness, and ductility rely on the ability of amorphous polymer chain segments for plastic flow at local level and the ability to response to an external load by absorption of energy rather than rupture. These abilities depend on molecular parameters like molar mass or entanglement density as well as external parameters like deformation rate and temperature; for semi-crystalline structures additional structural features such as tie molecules, or size and perfection of crystals and spherulites are important [52,53,54]. As far as we are aware, dedicated studies to assess the low-temperature-mobility of molecular chain segments in PA 11, as a requirement for superior mechanical behavior at such conditions, are not available, thus being subject of this work. The main idea to gain information about the mobility of chain segments at low temperature is to perform glass-annealing experiments on fully amorphous and semi-crystalline samples of well-defined vitrification and crystallization history and attempting to monitor changes of structure, which, as a prerequisite, requires chain mobility. As summarized above, such structural changes may include relaxations to decrease the free volume of the system or even ordering processes, which both are calorimetrically detectable by analysis of enthalpy-recovery or disordering peaks on heating the annealed system, respectively.

## 2. Materials and Methods

The study was performed using a biobased extrusion grade PA 11 Rilsan BESNO TL from Arkema (Colombes, France). Besides heat and light stabilizers, no further additives like nucleation agents or colorants are reported being present. The melt-volume rate is 1 cm^3^ /(10 min) (235 °C, 2.16 kg) [55] and the molar mass and polydispersity are about 17.2 kg/mol and 2, respectively [56]. The polymer was obtained as pellet. Independence of results obtained on the particular PA 11 BESNO TL grade is confirmed by qualitative analysis of a further Arkema PA 11 powder grade Rilsan T Naturelle BHV 2, allowing generalization of conclusions towards the entire PA 11 material family.

Thermal analysis of low-temperature changes of structure was done employing a power compensation fast scanning chip calorimeter (FSC) Flash DSC 1 from Mettler-Toledo (Greifensee, Switzerland). The instrument was coupled with a TC100 intracooler (Huber, Offenburg, Germany) to allow sub-ambient temperature operation and to assure high cooling-capacity, needed to subject samples to well-defined thermal histories including preparation of fully amorphous specimens. Note that the critical cooling rate to suppress any ordering of PA 11 macromolecules is around 1000 K/s, not achievable with conventional differential scanning calorimeters, which at best allow cooling at rates up to few hundreds of K/min [57,58]. As such, the temperature of the sample support in the FSC was controlled being −90 °C. Furthermore, the sample environment was purged with dry nitrogen gas using a flow rate of 40 mL/min. The empty FSC sensor, before loading the sample, was conditioned and temperature-corrected as described in the instrument operating instructions. PA 11 specimens for FSC analyses were prepared from the as-received pellets using a rotary microtome (Slee, Mainz, Germany) equipped with a tungsten carbide knife, to obtain rather artefact-free sections of about 10 µm thickness. These sections then were reduced in their lateral width to 50–100 µm under a stereomicroscope using a scalpel, to fit the central part of heatable area of the sensor, which was proven having a homogeneous temperature distribution [59]. The sample mass, as estimated from the heat-capacity increment on devitrification of fully amorphous preparations at *T*_g_, was between 100 and 160 ng. Reproducibility of experimental observations was assured by analysis of several, and independent of each other prepared samples, including the use of different sensors.

## 3. Results and Discussion

### 3.1. Glass Relaxation and/or Ordering of Fully Amorphous PA 11

**Figure 1:**
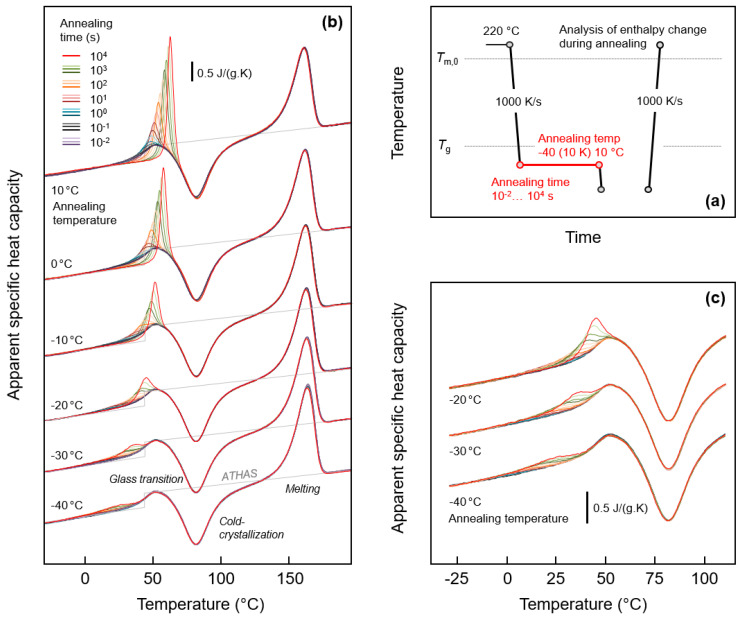
FSC analysis of glass relaxation and/or ordering of PA 11: (**a**) Temperature-time profile; (**b**) Sets of FSC heating scans, recorded at 1000 K/s, and obtained after annealing the glass of PA 11 at different temperatures for different time; (**c**) Enlargement of FSC heating curves obtained on PA 11 annealed at −20, −30, and −40 °C. Color-coding of curves in Figure 1c is in accordance with Figure 1b.

Figure 1a (top right) shows the temperature-time profile for analysis of possible relaxation and ordering of the glass of fully amorphous PA 11. The equilibrium liquid phase was cooled from 220 °C at a rate of 1000 K/s to below *T*_g_ of around 40 °C to different temperatures between -40 °C and 10 °C, and then the obtained glasses were annealed for different periods of time between 0.01 and 10,000 s. Structural changes occurring during annealing were analyzed by subsequent heating.

Figure 1b (left) is a plot of sets of FSC heating scans recorded after annealing the glass of PA 11 at different temperatures between −40 °C (bottom) and 10 °C (top). The different coloring of curves, as indicated in the legend, denotes the variation of the annealing time, and the gray line in each curve set represents the specific heat capacity of PA 11, as listed in the literature [19]. Heating of fully glassy PA 11 leads to devitrification of the glass at around 40 °C, detected with the step-like increase of the heat capacity. The glass transition is followed by exothermic cold-crystallization, which stretches from around 50 to 100 °C. Crystals formed on heating finally melt around and slightly above 150 °C. Note, though crystallization is suppressed on cooling the melt at a rate of 1000 K/s, heating at the same rate allows cold-crystallization due to formation of homogeneous crystal nuclei at temperatures below the cold-crystallization temperature, on both during cooling and heating. Most important in Figure 1b, however, are the observed endothermic peaks due to the annealing step, shown enlarged in Figure 1c (bottom right) for selected glass-annealing experiments performed at −20, −30, and −40 °C. Annealing at 10, 0, and −10 °C leads to observation of an enthalpy-recovery peak on heating, which superimposed to the main glass transition, and which increases in area with the time of annealing, as expected. Annealing at lower temperatures leads to a similar endothermic thermal event on heating, however, occurring well below the main devitrification process. For example, annealing at −40 °C causes a broad endothermic event, starting at around 0 °C and being well separated from the heat-capacity step due to the main glass transition. The detection of glass-annealing-caused endothermic peaks well below *T*_g_ serves at this point as main evidence for distinct mobility of chain segments 80 K below *T*_g_, as it proves structural reorganization even detectable by calorimetry. Further discussion about the specific changes of structure during annealing is provided in Section 3.3.

### 3.2. Glass Relaxation and/or Ordering in Semicrystalline PA 11

Since the presence of crystals may change the properties of the amorphous phase in polymers, for example when causing the formation of a rigid amorphous fraction (RAF) due to the covalent linkage of phases [60,61,62], the low-temperature mobility of chain segments in PA 11 may also be affected. For this reason, similar glass-annealing experiments as described above with Figure 1 were performed on partially crystallized samples.

**Figure 2:**
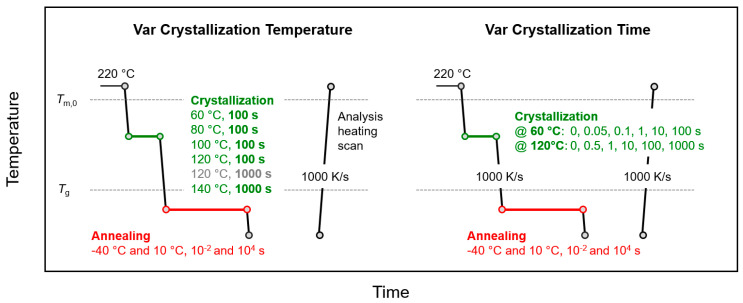
Temperature-time profiles for analysis of the effects of temperature (left) and time (right) of crystallization (green) on structural changes during annealing semicrystalline PA 11 below *T*_g_ (red).

The thermal protocol for crystallization and annealing semicrystalline samples below *T*_g_ is schematically shown in Figure 2, with the left and right parts representing experiments in which either the temperature or time of crystallization, respectively, were varied, as indicated (green segment).

After crystallization, the samples were rapidly cooled at a rate of 1000 K/s to annealing temperatures of 10 or −40 °C (red segment), before analysis of changes of structure by subsequent heating. In contrast to the experiments of Figure 1, for the sake of clarity of presentation, only annealing experiments with the samples annealed for 0.01 and 10,000 s are performed. The effects of variation of the crystallization temperature and time on annealing-induced changes of structure are presented in Section 3.2.1 and Section 3.2.2, respectively.

#### 3.2.1. Effect of Crystallization Temperature

**Figure 3:**
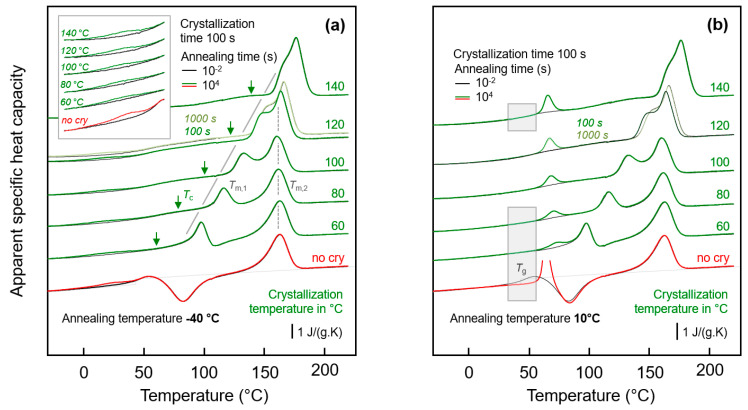
FSC analysis of glass relaxation and/or ordering in semi-crystalline PA 11: (**a**) FSC heating scans recorded at 1000 K/s after annealing semi-crystalline PA 11 at −40 °C; (**b**) FSC heating scans recorded at 1000 K/s after annealing semi-crystalline PA 11 at 10 °C. In both plots, the temperature of crystallization (*T*_c_) is indicated at the right-hand side of the curves. Black and green/red coloring of curves denote annealing times of 0.01 and 10,000 s, respectively, while red and green colors are used to highlight annealing experiments on fully amorphous (‘no cry’) and semi-crystalline samples, respectively, for easy comparison. *T*_m,1_ and *T*_m,2_ denote temperatures of melting of non-reorganized and reorganized crystals, respectively.

Figure 3a shows FSC heating scans obtained after annealing semi-crystalline PA 11 at −40 °C for 0.01 s (black curves) and 10,000 s (green/red curves). Note that curves obtained after annealing for 0.01 and 10,000 s strongly overlap, with differences only detected in the temperature range between about 0 and 50 °C, showing enlarged in the inset in the top left part. Crystallization was performed isothermally for 100 s at the indicated temperatures between 60 and 140 °C assuring for all samples a space-filling morphology; for comparison also the heating curves of an initially fully amorphous sample, denoted ‘no cry’ (red), is included. Motivation for analysis of samples crystallized at different temperatures is the formation of largely different semi-crystalline morphologies, possibly affecting the chain mobility in the neighbored amorphous phase. Figure 3b shows FSC heating scans in analogy to Figure 3a, however, with the various samples of different crystallization history annealed at 10 °C.

Heating fully amorphous samples, after annealing in the glassy state (see bottom curves in Figure 3a,b) reveals the glass transition close to 50 °C, which, on further heating, is followed by cold-crystallization and melting of crystals formed in the cold-crystallization event. Annealing for 0.01 s at both −40 and 10 °C does not lead to any detectable additional events, in contrast to annealing for 10,000 s. If annealing was performed at −40 °C then a small endothermic peak is observed between around 0 and 40 °C, while annealing at 10 °C leads during subsequent heating to the expected enthalpy-recovery peak at *T*_g_.

**Figure 4:**
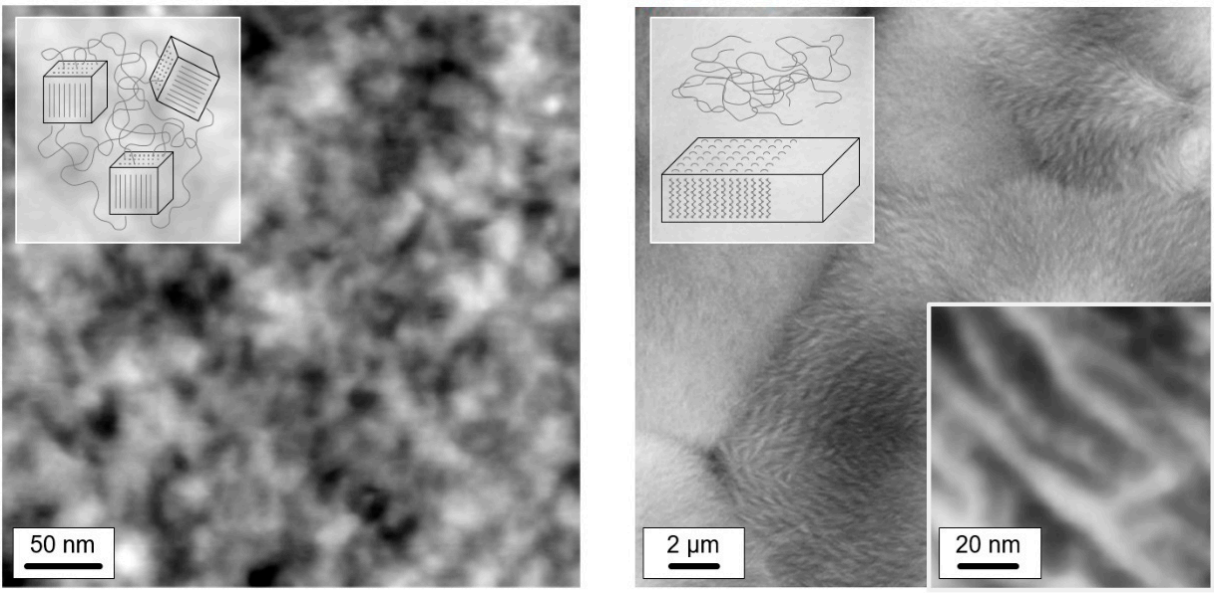
AFM height images of PA 11 crystallized at 60 °C (left) and 156 °C (right). The top left insets are sketches of the morphology of the crystals and their coupling with the surrounding amorphous structure. Reprinted/adapted from [68] (left) and [69] (right), with kind permission from Elsevier.

Heating scans obtained on semi-crystalline PA 11 reveal a qualitatively different behavior than was obtained with initially fully amorphous PA 11. Depending on the crystallization temperature (*T*_c_), melting of crystals at a temperature slightly higher than *T*_c_ (see gray line, *T*_m,1_) is detected, followed by a second melting event which is independent on *T*_c_ (see dashed gray line, *T*_m,2_). The peak at *T*_m,1_ is associated to melting of crystals which formed at *T*_c_ while the melting peak at *T*_m,2_ is associated to melting of crystals which reorganized during heating; detailed information about reorganization-related polymer double melting behavior is provided elsewhere [50,63,64,65,66]. Annealing at −40 °C (left plot), independent on the crystallization history, leads to a weak though highly reproducible endothermic event at around between 0 and 40 °C, similar as shown/discussed with Figure 1. In particular the shape of this peak, however, seems different for semi-crystalline and initially fully amorphous sample (compare red and green curves in the inset), as further discussed below, in Section 3.3. Though only by qualitative inspection, it appears that in case of PA 11 crystallized at rather low temperatures of, e.g., 60 or 80 °C the endothermic enthalpy-recovery- and/or disordering-peak is broadest. In case of annealing at 10 °C (right plot in Figure 3), the largest annealing-caused endothermic peak is detected for the non-crystalline sample (red) while in semi-crystalline samples (green) the area of the enthalpy-recovery peak is smaller, in particular if crystallization was performed at low temperatures. The reduced peak area of glass-annealing-caused peaks in heating scans of semi-crystalline samples annealed at 10 °C, compared to fully amorphous PA 11, straightforwardly may be explained by the lower amount of the amorphous fraction. The maximum crystallinity of PA 11 is around 30 % and is almost independent on the crystallization temperature [6,67]. This, however, implies that the decreasing peak area with decreasing Tc cannot be explained with a crystallinity-argument alone. Rather than it may be considered that the semi-crystalline morphologies and the possible rigid amorphous fraction strongly depend on *T*_c_. For demonstration, Figure 4 shows AFM images obtained on samples crystallized at 60 °C (left) [68] and at 156 °C (right) [69].

While high-temperature crystallization leads to formation of lamellae and spherulites, crystallization at temperatures lower than about 110 °C, due to the high nuclei density, yields small particle-like crystals with a much increased surface-to-volume ratio and large interfacial area with the surrounding amorphous phase, as further illustrated with the sketches in the top left insets in each AFM image. The large specific surface area of the small nodular crystals formed at 60 °C leads to severe mobility-constraints in the amorphous phase [70], which is evidenced by complete absence of a glass transition at the temperature where it occurs in the fully amorphous PA 11 (see lower gray-shaded box in the right plot of Figure 3). In case of spherulitic growth of lamellae, as occurring on crystallization at high temperature, a faint heat-capacity step due to devitrification of the amorphous phase is visible, pointing to a larger decoupling of the crystals from the amorphous structure when crystallizing at higher temperatures [71,72,73] (see upper gray-shaded box in the right plot of Figure 3). Similar trends regarding the effect of crystallization at different conditions on the glass transition of the amorphous phase are observed with the set of experiments presented in the left plot of Figure 3.

#### 3.2.2. Effect of Crystallization Time

**Figure 5:**
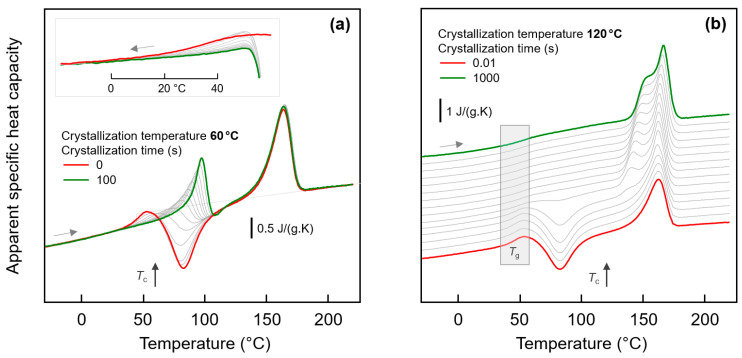
FSC analysis of isothermal melt-crystallization of PA 11: (**a**) FSC heating scans, recorded at 1000 K/s after melt-crystallization of PA 11 at 60 °C for different time between 0 (red curve) and 100 s (green curve). The gray curves were recorded after crystallization for 0.01, 0.02, 0.05, 0.1, 0.2, 0.5, 1, 2, 5, 10, 20, and 50 s. The inset show the glass transition at an enlarged scale; (**b**) FSC heating scans, recorded at 1000 K/s after melt-crystallization of PA 11 at 120 °C for different time between 0.01 (red curve) and 1000 s (green curve). The gray curves were recorded after crystallization for 0.02, 0.05, 0.2, 0.5, 1, 2, 5, 10, 20, 50, 100, 200, and 500 s.

In order to detect a possible effect of the fraction of crystals on the glass-annealing behavior of PA 11, the crystallization time was varied before the low-temperature annealing step. With the knowledge that different semi-crystalline morphologies form below and above about 110 °C (see Figure 4), crystallization experiments were performed at 60 and at 120 °C. Figure 5a,b show FSC heating scans of PA 11 recorded after melt-crystallization at 60 and 120 °C for different time, respectively. The crystallization time is increasing from bottom to top in the two sets of experiments, with the red and green curves obtained before and after completion of crystallization, respectively. In case of absence of crystals (red curves), there is detected the glass transition, cold-crystallization, and melting with increasing temperature, as expected for the selected heating rate of 1000 K/s; note that a heating rate >10,000 K/s is required to avoid cold-crystallization and, consequently, melting. With increasing time of crystallization, at both 60 and 120 °C, crystals grow. The formation of crystals is indicated in the heating scans with the appearance of a melting peak, which does not originate from crystals forming during heating by cold-crystallization. Melting of crystals isothermally forming at 60 and 120 °C occurs at a temperature slightly higher than *T*_c_, that is, below 100 and 150 °C, respectively. The crystallization-time-independent melting peak slightly above 150 °C is associated to crystals formed by melt-recrystallization.

Important in the context of the present study of the low-temperature glass-annealing behavior of PA 11, the FSC heating scans of Figure 5 reveal that presence of crystals forming at 60 and 120 °C differently affect the glass transition. While crystals growing at 120 °C lead to the expected decrease of the heat-capacity increment on devitrification the glass caused by the reduced amorphous fraction, without largely affecting *T*_g_, in case of crystallization at 60 °C, a glass transition cannot safely be detected. For demonstration, the inset in top left part of Figure 5a shows cooling scans after the isothermal crystallization, clearly showing that with increasing progress of crystallization the step-like change of the heat capacity due to vitrification of the remained amorphous phase gradually disappears. Note again that the maximum crystal fraction of PA 11, regardless the crystallization temperature, is, at best, 30 %, that is, an only minor change of the heat-capacity step on vitrification of the amorphous phase is expected, compared to the fully amorphous samples. It appears that the entire amorphous fraction freezes during crystallization at 60 °C.

**Figure 6:**
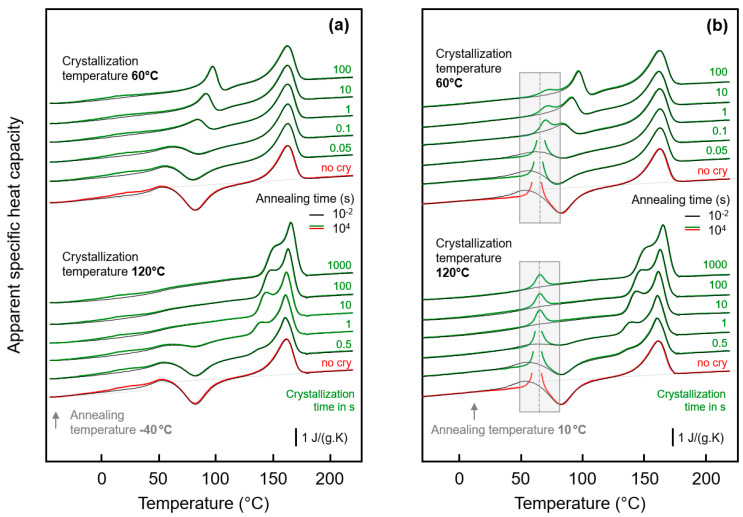
FSC analysis of glass relaxation and/or ordering in semi-crystalline PA 11: (**a**) FSC heating scans obtained after annealing semi-crystalline PA 11 at −40 °C; (**b**) FSC heating scans obtained after annealing semi-crystalline PA 11 at 10 °C. In both plots, the upper and lower sets of curves were obtained on samples crystallized at 60 and 120 °C, respectively, with the crystallization time indicated at the right-hand side of the curves. Black and green/red coloring of curves denote annealing times of 0.01 and 10,000 s, respectively, while red and green colors are used to highlight annealing experiments on fully amorphous (‘no cry’) and semi-crystalline samples.

The effect of the time of crystallization of PA 11 at 60 and 120 °C on the low-temperature annealing behavior at −40 and 10 °C is illustrated with Figure 6a,b, respectively. The plots show FSC heating scans after annealing PA 11 for 0.01 s (black curves) and 10,000 s (red/green curves), with the upper and lower sets of curves in each plot associated to prior crystallization at 60 and 120 °C, respectively. Within the various sets of curves, from bottom to top, the crystallization time is increasing, as indicated to the right of the curves. The thermal events occurring during heating an initially fully amorphous sample after low-temperature annealing have been discussed above with the bottom curves in Figure 3, and is therefore not repeated here; similar is true regarding the melting and reorganization behavior of crystals formed during crystallization. Inspection of the FSC heating scans in the left plot of Figure 6 leads to the main conclusion that low-temperature annealing at −40 °C causes structural changes in all samples of different crystal fraction (increasing with crystallization time) and different superstructure (as controlled by the crystallization temperature, see Figure 4). For all samples, a low-temperature endothermic peak is detected well below *T*_g_, proving enthalpy relaxation and/or ordering at the annealing temperature. While systematic and characteristic differences on annealing samples of different supermolecular structure at −40 °C cannot be detected with the applied analysis technique, the situation is different when annealing is performed at 10 °C. In agreement with the discussion of the effect of the crystallization temperature (see Figure 3), it is confirmed that crystallization of PA 11 at rather high temperature of 120 °C is connected with the observation of an temperature-position-wise crystallization-time-independent enthalpy-recovery peak on heating after prior annealing (see gray-shaded box in the lower set of curves). As expected, the area of the enthalpy-recovery peak decreases with increasing crystallization time, that is, with decreasing amount of amorphous fraction. Crystallization at 60 °C, in contrast, leads to a distinct immobilization of the amorphous phase as it is detected with absent devitrification at the glass transition temperature of fully amorphous PA 11, and consequently the enthalpy-recovery peak shifts to higher temperature (see gray-shaded box in the upper set of curves). Besides the temperature-shift, the enthalpy-recovery peaks are distinctly reduced in area suggesting reduced enthalpy relaxation during prior annealing at 10 °C.

### 3.3. Enthalpy Relaxation versus Crystallization/Ordering

Low-temperature annealing leads to endothermic peaks in FSC scans during subsequent heating. The nature of these peaks below *T*_g_ is not clear. These can be enthalpy-recovery peaks due to prior enthalpy relaxation/local-chain-relaxation processes within the relaxation spectrum [74], even at temperatures as low as 80 K below the main glass transition, as detected in the present work. Similar observation of sub-*T*_g_ enthalpy-recovery peaks is also reported for non-crystallizable polymers including poly (vinyl chloride) [75], polyarylate, polysulfone, and polycarbonate [76], bulk [77] and thin films of polystyrene [78,79], all discussed as presence of a different relaxation mechanism [80]. However, sub-*T*_g_-enthalpy-recovery peaks/presence of different relaxation mechanisms were also detected for metallic glasses [81,82,83,84,85], and small organic molecules, which form orientationally disordered crystals [86]. Though not being evidence, the frequent detection of annealing-caused endothermic sub-*T*_g_ peaks in non-crystallizable polymers [75,76,77,78,79] suggests that such peaks may not necessarily be associated to crystallization. However, endothermic sub-*T*_g_-peaks were also detected in amorphous and semi-crystalline poly (ethylene terephthalate), and discussed as both, being related to relaxation or ordering [87,88,89,90,91,92]. The latter process is described in the literature as concept of cohesional entanglement, involving “nematic interaction of neighboring chain segments” [89,90,91,92].

Considering the chain structure of PA 11, consisting of long aliphatic sequences with 10 methylene units, separated by amide groups, high chain mobility of intra-amide-group chain segments is expected. Mechanical and dielectric relaxation spectroscopy allowed identification of different dispersion regions [93,94,95]. Relaxation at about −120 °C was attributed to cooperative movement of methylene units between amide linkages (γ-dispersion). Evidence was provided by analysis of polyamides containing different number of methylene groups between the amide groups revealing a linear dependence of the area of the corresponding relaxation peak on the number of methylene units. Relaxation at around −50 °C (β-dispersion) is associated with segmental mobility involving amide groups not linked by hydrogen bonds with neighbored molecule segments. Both the γ- and β-dispersions are reported being independent on the crystallinity. The α-relaxation at about 40 °C, in contrast, represents the main glass transition and long-chain segmental motions in the mobile amorphous regions only. As such, it is assumed that annealing of PA 11 at −40 °C may preferably allow relaxation involving non-cooperative motion of methylene sequences and non-hydrogen-bond amide groups while annealing at 10 °C, that is, at a temperature close to *T*_g_, additionally leads to relaxation involving cooperative motion of larger units.

**Figure 7:**
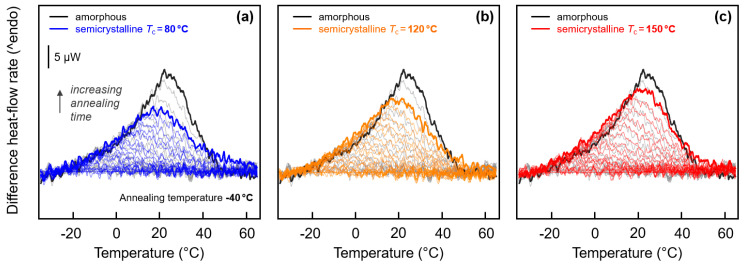
Difference FSC heat-flow rate heating curves, calculated from FSC heating scans (1000 K/s) obtained after annealing semi-crystalline PA 11 at −40 °C for different time by subtraction of the heating scan of non-annealed PA 11. The set of black/gray curves in each of the three plots was obtained on amorphous PA 11. Data sets shown in blue, orange, and red, in plots (**a**), (**b**), and (**c**), represent samples crystallized at 80, 120, and 150 °C, respectively. Curves obtained from samples annealed for 10,000 s are drawn bold.

**Figure 8:**
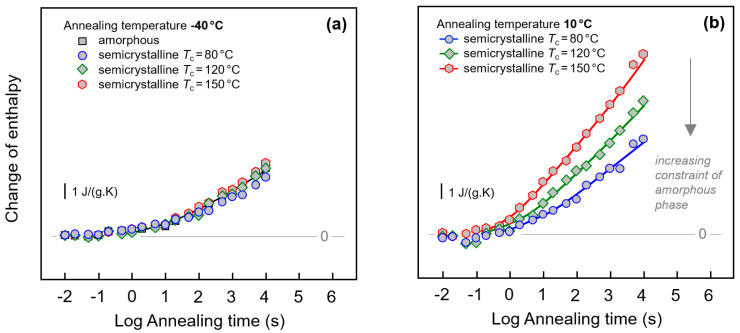
Change of enthalpy during annealing fully amorphous or semi-crystalline PA 11 at (**a**) −40 °C and (**b**) 10 °C. In case of semi-crystalline PA 11, crystallization was performed at 80, 120, and 150 °C, as indicated in the legends. Data are not normalized to the amorphous content in the various samples, as the crystallinity is almost independent on the crystallization temperature [6,67].

Quantitative analysis of the enthalpy-recovery peaks is provided with Figure 7 and Figure 8. Regarding low-temperature annealing at −40 °C, it has been outlined above, on discussion of Figure 3 (left), that the small enthalpy-recovery peak on heating appears being dependent on the structure. In case of the fully amorphous samples, the peak seems larger and narrower than in case of semi-crystalline samples (see again the inset in Figure 3a). This observation is reproducible and not related to any experimental uncertainty, despite effects are energetically small. In order to highlight the characteristic differences of the various enthalpy-recovery peaks obtained on samples of different initial structure, FSC difference-heating curves were calculated. For a given set of heating curves obtained after annealing a specific sample for different time at −40 °C, the scan obtained on the non-annealed sample was subtracted. Figure 7a–c show these difference-heating curves, with the coloring denoting the initial structure before annealing. The set of gray curves, shown in all plots as a reference, represents the initially fully amorphous sample, while blue, orange, and red colors in Figure 7a–c denote samples crystallized at 80, 120, and 150 °C, respectively. For easy comparison, data obtained on samples annealed for 10,000 s are drawn in bold.

The visual impression of an effect of the structure of PA 11 on inspection the enthalpy-recovery peak after annealing amorphous and semi-crystalline PA 11 at −40 °C (see inset in Figure 3a) is confirmed with Figure 7. Annealing amorphous PA 11 causes a rather sharp recovery-peak (gray/black curves) while in case of semi-crystalline PA 11 (blue, orange, and red curves) the peak is broader and less high. Moreover, there is observed a systematic effect of the crystallization temperature such that with decreasing crystallization temperature, and therefore increasing imperfection of the crystals, the peak height decreases, similar as was seen for the enthalpy-recovery peak after annealing at 10 °C (see Figure 3b). Most striking, annealing amorphous and semi-crystalline PA 11, crystallized at 150 °C (Figure 7c), apparently yield close-to-identical peak shapes and areas.

Quantitative information about the peak area/change of enthalpy during annealing PA 11 at −40 and 10 °C is provided with Figure 8a,b, respectively. As indicated in the legends, in case of semi-crystalline PA 11 crystallization was performed at 80, 120, and 150 °C, in order to identify the effect of different constraints of the amorphous phase when different supermolecular structures/semi-crystalline morphologies are evident. The change of enthalpy was calculated by integrating the FSC curves in the temperature range of the endothermic recovery-peak and subtracting from the obtained enthalpy-value the enthalpy of the non-annealed sample. Annealing at −40 °C leads to changes of structure beginning after about 0.1 s. The enthalpy then decreases steadily during annealing without reaching equilibrium within the pre-defined maximum annealing time of 10,000 s. Though it has been shown above with Figure 3a and Figure 7 that the initial structure of PA 11 affects the width and height of the recovery peak, the peak area seems only marginally dependent on the crystallization history of the sample. At best, a minor decrease of the relaxation strength is detected for the sample crystallized at 80 °C (blue circles).

In case of annealing PA 11 at 10 °C, discussed with Figure 8b, only data obtained on semi-crystalline samples are considered; we assume that overlapping enthalpy-recovery- and cold-crystallization peaks on heating complicate a reliable analysis of the annealing-caused enthalpy change (see also Figure 1, left, top set of curves). Relaxation after vitrifying the melt at 1000 K/s begins similarly as in case of the annealing experiment at −40 °C after about 0.1 s, however, the kinetics of the enthalpy change depends now strongly on the supermolecular structure. Within the limit of the maximum annealing time, the largest enthalpy change is observed for PA 11 crystallized at 150 °C. In case of PA 11 crystallized at lower temperatures of 120 and 80 °C, relaxation is slower, thus yielding a smaller enthalpy change after a given annealing time.

The α-relaxation at 10 °C only occurs in the mobile amorphous phase. Reduced amount of amorphous phase, when comparing fully amorphous and semi-crystalline PA 11, leads therefore to the expectation of a lowered maximum relaxation strength, that is, a lower final value of the enthalpy change when steady state is reached; unfortunately, the maximum annealing time was too short to confirm this expectation. Though it also may be speculated that reduced amount of amorphous structure leads to a reduction of the overall slope of the curves, a major effect is only seen in annealing experiments performed at 10 °C. The data are interpreted such that in case of PA 11 crystallized at 150 °C the amorphous phase exhibits similar structure and segmental mobility as fully amorphous PA 11. However, if crystallization is performed at lower temperatures, then increasing covalent coupling of the crystalline and amorphous phases as well as increasing interfacial area due to smaller crystals may cause mobility constraints (see Figure 4) which then significantly affect the relaxation kinetics. Such mobility constraints have been detected by the changed glass transition behavior, discussed above, e.g., with Figure 5 and Figure 6. Since relaxation at −40 °C is assumed involving mainly non-cooperative conformational changes, such constraints are of only minor influence regarding the relaxation kinetics.

**Figure 9:**
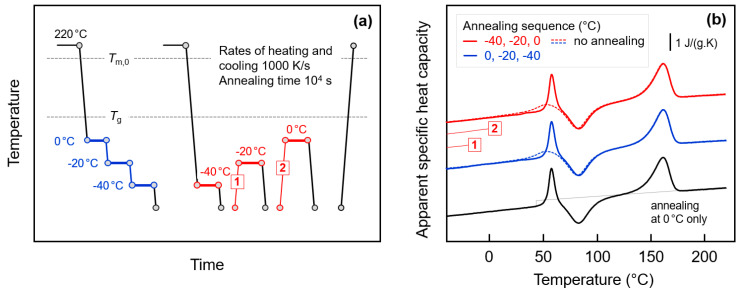
FSC analysis of glass relaxation and/or ordering in fully amorphous PA 11 by sequential annealing: (**a**) Temperature-time protocol for glass-annealing experiments with successively decreasing (blue) or increasing (red) annealing temperatures. (**b**) FSC heating scans obtained after annealing the glass of PA 11, in the given sequence, at 0, −20, and −40 °C (blue), and −40, −20, and 0 °C (red), for 10,000 s (solid lines). The dashed lines represent data obtained on non-annealed samples, for comparison. The lower curve (black) was obtained on a sample annealed at 0 °C only.

The above discussion was based on the assumption that the endothermic annealing-caused peaks in the FSC heating scans are caused by enthalpy relaxation of the amorphous phase, that is, by rearrangement of molecular segments involving conformational changes, leading to a decrease of the free volume. Crystallization, that is, formation of small domains consisting of few parallel aligned short chain segments, separated from amorphous structure by a phase boundary, would lead to a similar decrease of the enthalpy. The endothermic peaks observed on subsequent heating then need being interpreted as melting or disordering peaks. In order to prove/disprove glass-crystallization in the experiments discussed above, the annealing conditions were tailored as described with Figure 9. Figure 9a shows the temperature-time protocol for glass-annealing experiments with successively decreasing (blue) or increasing (red) annealing temperatures, before analysis of the change of structure with the final heating scan. Figure 9b is a plot of the final FSC heating scans obtained after annealing the glass, with the color-coding corresponding to that of the thermal profiles in Figure 9a. The dashed lines represent data obtained on non-annealed samples, and the lower curve (black) was obtained on a sample annealed at 0 °C only. The two thin red lines, labeled ‘1′ and ‘2′, illustrate specific transfers of the sample between different annealing steps as indicated in Figure 9a.

The main idea of the glass-annealing experiment with successively decreasing temperature is the presumption that crystallization at low temperature, e.g., at −40 °C, cannot be suppressed by prior annealing at higher temperature. Such behavior is reasonable since with decreasing supercooling of the melt the critical size of nuclei decreases [96] and since even after crystallization at high temperature to a maximum extent, a large fraction of amorphous phase remains. It has been shown above that even in samples crystallized to a maximum extent at higher temperature, that is, well above *T*_g_, annealing the glass at −40 °C leads to observation of endothermic peaks on subsequent heating. If these were melting/disordering peaks, then crystals grew at −40 °C independent on prior crystallization at higher temperature. In such case, however, annealing at 0 °C and −20 °C would not significantly change the crystallization process at −40 °C and on heating still endothermic melting/disordering should be expected. However, the experiment of Figure 9 disproves such model as glass-annealing at −40 °C after prior annealing at 0 and −20 °C does not lead to observation of endothermic sub-*T*_g_ events on subsequent heating, but only the observation of the classical enthalpy-recovery peak at *T*_g_ (see blue solid line in Figure 9b). Obviously, successive annealing at 0 °C (and −20 °C) allows relaxation of the glass to an extent not requiring further relaxation at lower temperature of −40 °C, as proven by comparison with the behavior of a sample which was annealed at 0 °C only (black curve). As such, the glass-annealing experiment with successively decreasing temperature supports the notion that endothermic peaks on heating after single-step annealing experiments are due to the relaxations but not crystallization/ordering.

Further support of this suggestion is provided by the glass-annealing experiment with successively increasing annealing temperature. Here we follow the idea for distinguishing relaxation on one side and crystallization/ordering on the other side by largely different memory effects after endothermic enthalpy-recovery or melting/disordering, respectively. While after enthalpy-recovery the structural state of the amorphous phase is recovered, after melting/disordering a non-fully randomized melt, that is, so-called self-nuclei [49,65,68,69], may exist which during further annealing would promote renewed crystallization and ordering, and consequently lead to observation of melting/disordering at higher temperature. However, such melting/disordering peaks at successively higher temperature are not detected but only the enthalpy-recovery peak at *T*_g_, thus excluding glass-crystallization/ordering as origin of the endothermic sub-*T*_g_ peaks on subsequent heating.

## 4. Conclusions

Application of FSC allowed preparation of PA 11 samples of largely different supermolecular structure. Considering the rather high critical cooling rate of this polymer of 500–1000 K/s to suppress crystallization during cooling the melt and to obtain a crystal-free glass, alternative methods are not available. Moreover, the high cooling capacity of the used instrumentation allowed crystallization of the melt at well-defined supercooling conditions, and generation of qualitatively different semi-crystalline morphologies (see Figure 4).

Fully amorphous PA 11 and PA 11 of different semi-crystalline morphology were then annealed at different temperatures well below *T*_g_, with the main purpose to calorimetrically prove/disprove mobility of chain segments at temperatures as low as −40 °C (≈*T*_g_ − 80 K) where excellent impact resistance is. It was found that annealing PA 11 at such low temperature, regardless the thermal history and supermolecular structure including crystallinity as well as crystal shape and size, permits distinct enthalpy relaxation at rather short time scale with the structural changes reverting on subsequent heating as detected with pronounced sub-*T*_g_-enthalpy-recovery peaks. Enthalpy relaxation during annealing at temperatures only slightly lower than *T*_g_ is sensitive to the physical structure of PA 11 as it was shown that with increasing coupling of the crystalline and amorphous phases cooperative rearrangements at the length scale of the α-relaxation slow down. The latter observation paralleled the detection of an increase of *T*_g_ in case of presence of small nodular crystals compared to systems containing large lamellar or no crystals where the amorphous phase is less constrained. The pronounced dependence of the enthalpy relaxation and consequently the reduction of entropy at temperatures slightly below *T*_g_ on the semi-crystalline morphology and, contrary, the rather weak dependence on morphology of both quantities for low temperature annealing at *T*_g_ – 80 K indicate different molecular mechanisms for the processes occurring in both temperature ranges. The latter even seems proceeding also in the crystalline fraction of the material as shown by nearly equal effects in the amorphous and semi-crystalline PA 11. The present study revealed for the first time annealing-caused sub-*T*_g_-enthalpy-recovery peaks in semi-crystalline polyamide, pointing to universality of such observation when considering its detection in a large variety of different structures.
